# IL-15 in the Combination Immunotherapy of Cancer

**DOI:** 10.3389/fimmu.2020.00868

**Published:** 2020-05-19

**Authors:** Thomas A. Waldmann, Sigrid Dubois, Milos D. Miljkovic, Kevin C. Conlon

**Affiliations:** Lymphoid Malignancies Branch, Center for Cancer Research, National Cancer Institute, National Institutes of Health, Bethesda, MD, United States

**Keywords:** interleukin-15, natural killer cells, CD8 T cells, immunotherapy of cancer, immunological checkpoints

## Abstract

We completed clinical trials of rhIL-15 by bolus, subcutaneous, and continuous intravenous infusions (CIV). IL-15 administered by CIV at 2 mcg/kg/day yielded a 38-fold increase in 10- day number of circulating NK cells, a 358-fold increase in CD56^bright^ NK cells and a 5.8-fold increase in CD8 T cells. However, IL-15 preparations administered as monotherapy were ineffective, due to actions of immunological checkpoints and due to the lack of tumor specific targeting by NK cells. To circumvent checkpoints, trials of IL-15 in combination with other anticancer agents were initiated. Tumor-bearing mice receiving IL-15 with antibodies to CTLA-4 and PD-L1 manifested marked prolongation of survival compared to mice receiving IL-15 with either agent alone. In translation, a phase I trial was initiated involving IL-15 (rhIL-15), nivolumab and ipilimumab in patients with malignancy (NCT03388632). In rhesus macaques CIV IL-15 at 20 μg/kg/day for 10 days led to an 80-fold increase in number of circulating effector memory CD8 T cells. However, administration of γc cytokines such as IL-15 led to paralysis/depression of CD4 T-cells that was mediated through transient expression of SOCS3 that inhibited the STAT5 signaling pathway. This lost CD4 helper role could be restored alternatively by CD40 agonists. In the TRAMP-C2 prostate tumor model the combination of IL-15 with agonistic anti-CD40 produced additive effects in terms of numbers of TRAMP-C2 tumor specific Spas/SCNC/9H tetramer positive CD8 T cells expressed and tumor responses. A clinical trial is being initiated for patients with cancer using an intralesional anti-CD40 in combination with CIV rhIL-15. To translate IL-15-mediated increases in NK cells, we investigated combination therapy of IL-15 with anticancer monoclonal antibodies including rituximab in mouse models of EL-4 lymphoma transfected with human CD20 and with alemtuzumab (CAMPATH-1H) in a xenograft model of adult T cell leukemia (ATL). IL-15 enhanced the ADCC and therapeutic efficacy of both antibodies. These results provided the scientific basis for trials of IL-15 combined with alemtuzumab (anti-CD52) for patients with ATL (NCT02689453), with obinutuzumab (anti-CD20) for patients with CLL (NCT03759184), and with avelumab (anti-PD-L1) in patients with T-cell lymphoma (NCT03905135) and renal cancer (NCT04150562). In the first trial, there was elimination of circulating ATL and CLL leukemic cells in select patients.

## Introduction

The goal of immunotherapy is to direct the host immune system to attack patients' cancer (1, 2). Clinical trials initially focused on efforts to enhance immune responses using the stimulatory cytokines IFNα or IL-2 (2). High-dose IL-2 was approved by the FDA for the treatment of patients with metastatic melanoma and metastatic renal carcinoma (3) but caused severe systemic toxicity, including capillary leak syndrome, hypotension, hypoxia, and oliguric renal failure. These problems prompted the investigation transition to IL-15 in an effort to obtain the benefits of IL-2 but with fewer adverse events (AEs) (2).

IL-15 was identified by our group and by Grabstein in culture supernatants from HUT102 and Cv1/EBNA cell lines that stimulated proliferation of the cytokine dependent T-cell line CTLL-2 (4–6). IL-15 is a 14–15 kDa 4 alpha-helix-bundle family cytokine family member that stimulates the generation of NK, NKT, gamma delta, IL/C1, intraepithelial lymphocytes, innate cells expressing CD103+ CD56+ CD44+ and memory CD8 T cells (2, 7–17). Some IL-15 regulation of protein production occurs at the level of transcription; however, most control is at translation (18). Type I and II interferons, CD40 ligation, and Toll-like receptor stimuli stimulate transcription (19). IL-15 translation is impeded by multiple 5′-untranslated region (UTR) AUG sequences, a long signal peptide and a negative regulatory element in the coding sequence C-terminus (19). IL-15 mRNA is expressed by many tissues. However, IL-15 protein is largely limited to dendritic cells, macrophages, and monocytes (20). IL-15 signals through a heterotrimeric receptor that is composed of the common gamma chain (γc) subunit (CD132) shared with IL-2, IL-4, IL-7, IL-9, and IL-21; the beta chain (βc) subunit (IL-2/IL-15R, CD122) shared with the IL-2 receptor and a private IL-15 specific alpha subunit IL-15Rα (CD215) (2, 8, 20, 21). IL-15 binding to the IL-2/IL-15Rβ/γc heterodimeric receptor induces JAK1 activation that phosphorylates STAT3 via the beta chain, and JAK3 activation that phosphorylates STAT5 via the gamma chain (20–25).

IL-15, like IL-2, stimulated proliferation of T cells, induced generation of cytotoxic lymphocytes and memory phenotype CD8 T cells, and stimulated proliferation and maintenance of natural killer (NK) cells (2, 8, 20). In contrast to IL-2, IL-15 did not mediate activation-induced cell death (AICD), did not consistently activate Tregs and caused less capillary leak syndrome (2, 8, 26). IL-2 is a promiscuously secreted molecule, whereas IL-15 is locally secreted in small quantities where membrane-bound IL-15 induces signals at an immunological synapse (27–32). IL-15 and IL-15Rα co-expressed by monocytes and DCs become associated on cell surfaces where IL-15 is presented *in trans* to NK and CD8 memory T-cells (27–32). In addition, IL-15 cis presentation is required for optimal NK-cell activation in lipopolysaccharide-mediated inflammatory conditions (33).

Although IL-2 stimulates immune responses directed at cancer cells, it also suppresses immune responses by maintenance of CD25^+^ Foxp3 T-regulatory cells and by participation in AICD (34–37).

Efficacy was observed with IL-15 in multiple murine immunotherapy trials including the syngeneic TRAMP (transgene adenocarcinoma mouse prostate) -C2 prostatic cancer, Pme1-1, B16 melanoma, MC38 and CT26 colon carcinoma models suggesting that IL-15 might be more effective than IL-2 in cancer therapy (38–40). Ten-day 20 mcg/kg/day administration of IL-15 to rhesus macaques by continuous infusion (CIV) was associated with an 80–100 fold increase in the number of circulating effector memory CD8 T cells (41, 42). To translate the observation of the effect of IL-15 on NK cells and CD8 cells, we have completed first-in-human trials of rhIL-15 by bolus, subcutaneous and continuous intravenous infusions (CIV) (2, 43–45). However, IL-15 administered as monotherapy was ineffective, likely due to the actions of immunological checkpoints (2). To circumvent such checkpoints, trials of IL-15 in combination with other anticancer agents have been initiated and are a major focus of this review.

## Clinical Trials Using IL-15 in the Treatment of Cancer

We initiated a first-in-human phase I trial of recombinant *Escherichia coli* produced IL-15 administered by IV bolus daily for 12 days to patients with metastatic malignancy (2, 43) (Table 1). The initial dose of 3 μg/kg/day was too toxic with patients developing grade 3 thrombocytopenia and hypotension, and doses of 1.0 and 0.3 μg/kg/day were added (2, 43). All patients at the 0.3 μg/kg dose level received 12 doses without dose-limiting toxicity (DLT). With the 3 μg/kg dose level as assessed by flow cytometry there was a 10-fold increase in the circulating NK numbers, a 3-fold increase in the number of CD4 cells and an 8-fold increase in the number of CD8 T cells. Stable disease was the best response. Inflammatory cytokines IL-6 and IFN-γ were markedly elevated (50-fold), a phenomenon which coincided with acute clinical toxicities of fever, chills and blood pressure changes. To reduce toxicity by reducing C_max_ excess, mediated cytokine release, and macrophage activation syndrome, two additional clinical trials were initiated, one by subcutaneous, and another by continuous intravenous infusion (2, 44, 45).

**Table 1:** IL-15 Clinical trials in patients with metastatic malignancy.

**IL-15 agent**	**MTD or expansion dose/dosing schedule**	**Study population**	**Serious and notable adverse events**	**Maximum fold increase in total NK cells at MTD**	**Maximum fold increase in CD56 bright NK cells**	**Maximum fold increase in CD8 T cells**	**Best clinical Response**	**References**
*E. coli* rhIL-15	0.3 μg/kg/d bolus i.v. 12 consecutive days	18 patients with malignant melanoma or renal cell cancer	Grade 3 hypotension Grade 3 thrombocytopenia Grade 3 ALT, AST elevations	2–3	3–4	3	Stable disease (5 patients had 10–30% decrease in marker lesions and 2 disappearance of lung lesions)	Conlon et al. (43) National Cancer Institute, NIH
*E. coli* rhIL-15	2 μg/kg/d CIV for 10 days	27 patients with metastatic solid tumors	2 deaths (one due to gastrointestinal ischemia and one due to disease progression) Grade 3 bleeding Grade 3 papilledema Grade 3 uveitis Grade 3 hepatic encephalopathy	38	358	5.8	Stable disease	Conlon et al. (45) National Cancer Institute, NIH
*E. coli* rhIL-15	2 μg/kg/d SC days 1–5, 8–12	19 patients with advanced solid tumors	Grade 2 pancreatitis Grade 3 cardiac/chest pain	10.8	39.7	3.3	Stable disease	Miller et al. (44) Minnesota Cancer Center
ALT-803	10 μg/kg IV or SC weekly for 4 weeks	33 patients with hematological malignancies	2 deaths (one due to sepsis, one due to intracranial hemorrhage) Grade 4 sepsis Grade 2 pemphigus	8	8	2	1 CR, 1 PR, 3 SD	Romee et al. (46) Minnesota Cancer Center
ALT-803	20 μg/kg SC 4 consecutive weeks every 6 weeks	21 patients 11 IV, 13 SC with solid tumors	Grade 4 congestive heart failure Grade 4 neutropenia Injection site reaction	3.3	6.3	6.3	No PR or CR	Margolin et al. (47) Fred Hutchinson Cancer Center

*CIV, continuous intravenous infusion; d, day; IL-15, interleukin 15; IV, intravenous; Kg, kilogram; NA, not available; SC, subcutaneous; CR, complete response; PR, partial response; SD, stable disease; CRS, cytokine release syndrome*.

In the subcutaneous rhIL-15 trial in refractory solid tumor cancer patients' therapy consisted of daily (Monday–Friday) subcutaneous injections of rhIL-15 for 2 consecutive weeks (44). Nineteen patients were treated with rhIL-15. Among 19 patients treated there were two serious events: grade 2 pancreatitis, grade 3 cardiac chest pain, hypotension, and elevated troponin. No objective responses were observed. Treatment induced a 3-fold increase in the number of circulating CD8 T cells, a 10.8-fold expansion of circulating NK cells, and a 39.7-fold increase in CD56^bright^ cells.

In an additional trial 27 patients were treated for 10 days by continuous intravenous infusion with rhIL-15; with 2.0 μg/kg/day identified as the MTD (45). There were eight serious adverse events including: papilledema, uveitis, pneumonitis, duodenal erosions, two bleeding events, and two deaths, one likely due to drug-related gastrointestinal ischemia (2, 45). Limited reduction in tumor volume was observed in several patients, but stable disease per RECIST 1.1 criteria was the best response noted (45). In this trial, the IL-15 C_max_ was at 48 h, followed by a decline of serum IL-15 concentrations during the infusions to 8% of the maximum level by days 8–10 of infusion. This decline may reflect IL-15-mediated induction of the number of IL-15 receptor-bearing cells with an increase in the number of IL-2/IL-15Rβ (CD122) receptors per cell acting as a sink binding the infused rhIL-15. There was a mean 5.8-fold increase in the number of circulating CD8 T cells, a 38-fold increase in the total of NK cells and a 358-fold increase in CD56^bright^ NK cells (2, 45).

Studying purified NK cells *in vitro* Felices et al. (48) suggested that continuous treatment with IL-15 exhausts purified NK cells resulting in decreased viability and a cell cycle arrest gene expression pattern. Furthermore, they propose that their findings should inform IL-15 dosing strategies (2, 48). Our studies with IL-15 *in vivo* by CIV to humans do not support these conclusions. The proliferation rates of different subsets of NK cells 2 days after the termination of 10-day IL-15 CIV assayed by Ki-67 were over 90% (45, 49). The cytolytic capacities were very effective for both CD56^dim^ and CD56^bright^ NK subsets. At the maximum NK level 2 days following the termination of IL-15 CIV the antibody-dependent cellular cytotoxicity (ADCC) assayed with CD20 antibody-coated Raji cells, natural cytotoxicity to K-562 cells mediated by NKp30, NKp46, and MICA/NKG2D mediated cytotoxicity was exceptionally effective (2, 49). These observations on the effects of IL-15 on NK subsets do not support the hypothesis that such strategies would be associated with NK-cell exhaustion but rather support the view that after 10-day rhIL-15 CIV NK cells remain effective.

**Figure 1:**
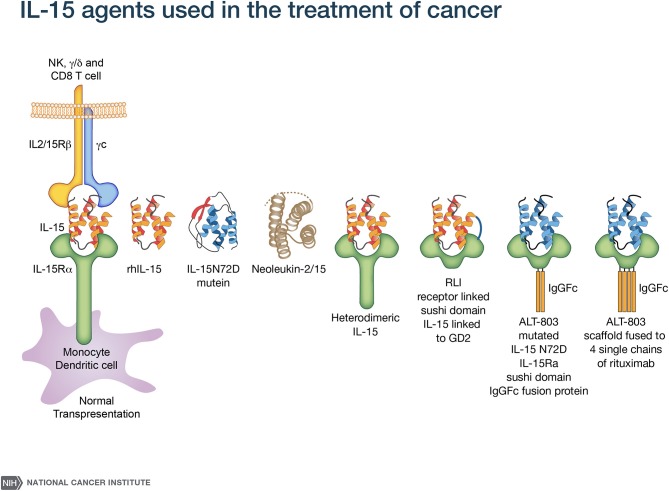
IL-15 agonists used in immunotherapy. IL-15 preparations in clinical use include rhIL-15 produced in *Escherichia coli* (43–45), an IL-15N72D mutein (50), heterodimeric mammalian IL-15 (hetIL-15) (51–54), RLI, a fusion protein consisting of IL-15 linked to the cytokine-binding (sushi) domain of IL-15R alpha (59). Anti-CD-20-RLI and anti-GD2-RLI are fusion proteins consisting of RLI linked to anti-CD20 or anti-GD2, respectively (55, 61). ALT-803 (Altor Pharmaceutical) represents a mutated N72D) IL-15 (asparagine replacing aspartic residue) linked to the sushi domain of IL-15R that is fused to an IgG-Fc fragment to increase *in vivo* survival (56, 57) and ALT-803 scaffold has been fused to 4 single- chains of the tumor-targeting monoclonal antibody rituximab (58).

A major challenge with rhIL-15 is that it has a short *in vivo* survival. Therefore, an array of alternative IL-15 agents associated with IL-15Rα were introduced clinically (50–61) (Figure 1). These included an IL-15N72D mutein with a 4-5-fold increase in biological activity, heterodimeric mammalian IL-15/IL-15Rα (hetIL-15) (51–54) a heterodimer consisting of IL-15 and IL-15Rα (51–54), the RLI, a fusion protein consisting of IL-15 linked to the cytokine binding (sushi) domain of IL-15Rα (59, 60), RLI-anti-CD20 and RLI-CD20 which are RLI linked to anti-CD20 or GD2 (55, 61), ALT-803 a mutated (N72D) IL-15 linked to the sushi domain of IL-15Rα that is fused to an IgG-Fc fragment to increase *in vivo* survival (55–57), the ALT-803 scaffold fused to 4-single chains of rituximab, a tumor-targeting monoclonal antibody (2, 58). ALT-803 was administered to 33 patients with hematological malignancies via IV or SC once weekly for 4 doses and pharmacokinetic analysis showed prolonged serum concentrations following SC compared to IV infusion (46). There were 2 deaths–one due to sepsis, and one intracranial hemorrhage purported to be unrelated to ALT-803. Administrations of hetIL-15 or ALT-803 by subcutaneous injection produced concentric injection site reactions up to 30 cm in diameter erythematous plaques that were associated with infiltration of CD56+ NKp46^−^ γδ T-cells. This and other systemic AEs with ALT-803 and hetIL-15 precluded further increases in doses of IL-15 bearing agents. When the maximum fold increases in the number of circulating NK cells with different agents and dosing schedules were compared, rhIL-15 administered by bolus infusion at the MTD (0.3 mcg/kg/day) yielded a 2–3 fold increase in NK cells (43) (Table 1). rhIL-15 administered subcutaneously at the expansion dose of 2 mcg/kg/day on days 1–5, and 8–12 was associated with a 10.8-fold increase in total circulating NK cells and a 39.7-fold increase in CD56^bright^ NK cells (44). The ALT-803 mutant at 10 mcg/kg/week elicited an 8-fold increase in NK cells (46). rhIL-15 by CIV at 2 mcg/kg/day for 10 days resulted in the greatest increase with a 38-fold increase in circulating total NK cells and a 358-fold increase in CD56^bright^ NK cells (45).

Novel approaches with IL-15 are being developed to yield the desired pharmacokinetics of IL-15 plus IL-15Rα with one dosing per week along with maximal increases in NK and CD8 T cells provided by CIV rhIL-15. PEGylated IL-15 to prolong survival is being developed. Furthermore, a long-acting rhIL-15 depo for enhanced cancer immunotherapy is being developed, with IL-15 mixed with an aqueous solution of PLGA-PEG, a copolymer that is in solution at room temperature but transitions into a hydrogel at body temperature (Tan and Waldmann unpublished observations).

## IL-15 in Combination Therapy

### IL-15 and Haploidentical Natural Killer Cell Therapy for Advanced Acute Myeloid Leukemia

Although rhIL-15 by CIV yielded dramatic augmentation in the number of circulating NK cells, it will have to be used in combination with other anticancer agents due to the inhibitory actions of immunological checkpoints and the lack of tumor specific targeting by NK cells (Tables 2, 3). A major challenge in IL-15 immunotherapy is finding a combination of drugs with new mechanisms of action that improve the outcome achieved with the existing standard of care and simultaneously result in fewer toxic effects for patients. Forty-two patients with refractory acute myeloid leukemia received intravenous (IV) (NCT01385423) or subcutaneous (SC) (NCT02395822) recombinant human IL-15 (rhIL-15) after lymphodepleting chemotherapy and haploidentical NK cell infusions (75). Escalating doses of rhIL-15 (0.3–1.0 mcg/kg) were given on 12 consecutive days in a phase I trial to 26 patients. Subcutaneous IL-15 at 2.0 mcg/kg was administered in a phase II trial to 16 patients. With the IV dosing and dose level 3 (1 mcg/kg) dose-limiting toxicity consisting of grade 4 pulmonary toxicity (diffuse alveolar hemorrhage) in one patient and prolonged neutropenia (beyond 42 days) in 2 of 4 patients at this dose were observed. In the subsequent phase II trial using 2 mcg/kg SC of rhIL-15 there was a previously unreported cytokine release syndrome (CRS) observed in 56% of patients with concurrent neurological toxicity in 5 of 9 patients that was responsive to steroid and tocilizumab treatment. Eight of 25 evaluable patients receiving IV IL-15 had a response with 6 CRs and 2 CRis. The overall response to SC IL-15 was 6 of 15 patients with a CR in one and CRi in 5 (75). Thus, haploidentical NK cell infusions given with rhIL-15 achieved remissions in about 35% of patients with refractory acute myeloid leukemia.

**Table 2:** Preclinical studies of IL-15 IN combination immunotherapy and cancer.

**IL-15 agent**	**Combination agent**	**Study**	**Best response to IL-15**	**Reference**
rhIL-15	Rituximab	Mouse graft EL4 transfected with human CD20	Prolongation of survival increase in ADCC	Zhang et al. (62) National Cancer Institute, NIH
rhIL-15	Alemtuzumab	Mouse xenograft with ATL cell line	Prolongation of survival increase in ADCC	Zhang et al. (62) National Cancer Institute, NIH
RLI		HCT human colon carcinoma B16F10	NK mediated reduce tumor growth overcoming limited effect of IL-15	Bessard et al. (60)
RLI anti-GD2		SC EL4, metastatic N x S2 Neuroblastoma	Better murine survival than anti-G2D or RLI alone	Vincent et al. (61)
RLI anti-CD20		Human B cell lymphoma in SCID mice	Prolonged survival of mice beyond that of RLI or anti-CD20 alone	Vincent et al. (55)
rhIL-15	Cetuximab	Triple negative breast cancer cell line EGFR expression Hbbr with KRAS mutation 50:1 effector target	Increase in TBMC, ADCC against cell lines from 28 to 34% without IL-15 to 71% with this increase in NK expression and activation of receptors	Roberti et al. (63) Centro de Investigaciones Oncológicas, Buenos Aires, Argentina
ALT-803	Anti-CD20	Primary human B cell lymphoma and B cell lines. Two human NK xenografts in NOD/SCID mice	Significant increase in degranulation, IFNα production, decrease in tumor cells, and ADCC by human NK cells against B cell lymphoma. Increase mouse survival.	Rosario et al. (64) Johns Hopkins Medicine, Baltimore, Maryland
IL-15	Rituximab	CLL cells γc^−/−^ mice	Enhanced cytotoxicity against CLL cells with overcome TGFβ mediated immunosuppression.	Moga et al. (65) Department of Immunology Hospital Santa Creu i Sant Pau, Barcelona, Spain
ALT-803 fused to Rituximab 2B8T2M		B-cell lymphoma cells xenograft to SCID/NOD mice	2B8T2M better cytokines, better survival of mice with xenografts better depletion of B cells in monkeys.	Liu et al. (66) Alto BioScience Corp, Miramar, Florida
rhIL-15 0.25 mcg/day daily 5x/week for 4 weeks	Anti-CD40	TRAMP-C2 graft in mice	Prolongation of survival of mice with xenograft. Development of tumor specific CD8 T cells.	Zhang et al. (67, 68) National Cancer Institute, NIH
ALT-803	Anti-gp75, TA99 anti-PD-L1	Mice bearing B16F10	Prolong survival through activation of NK cells and expansion of CD8^+^CD44^high^ T cells. Addition of anti-PD-L1 further increases antitumor activity.	Chen et al. (56) University of South Carolina Medical School
TriKE bispecific NK cell engaged against CD16 modified IL-15 crosslinker.		Cr51 release degranulation vs. carcinoma cell lines.	TriKE with IL-15 when compared to BiKE without IL-15 showed enhanced ADCC with improved activation and survival of NK cells.	Schmohl et al. (58) University of Minnesota Medical School
rhIL-15	Anti-CTLA-4 Anti-PD-L1	Mouse TRAMP-C2 prostate, CT26 colon carcinoma models.	Simultaneous inhibition of two regulatory 7-cell inhibitory checkpoints enhanced IL-15 efficacy in murine tumor models.	Yu et al. (69, 70) National Cancer Institute, NIH
IL-15 sIL-15Rα/Fc	Anti-PD-1	HT-29 xenograft in NOD/SCID mice.	Tumor growth inhibition.	Zhao et al. (71) Shanghai University
IL-15/IL-15Rα armed oncolytic virus	Anti-PD-1	MC38 colon mouse carcinoma or ID8 ovarian cancer models.	CD8 T cell mediated by IL-15 armed oncolytic virus. Antitumor immunity was dramatically improved by addition of anti-PD-1	Kowalsky et al. (72) University of Pittsburgh School of Medicine
ALT-803 (N-803)	Anti-PD-L1	4TI Triple negative breast and MC38 colon tumor bearing mice	ALT-803 enhanced anti-PD-L1 antitumor efficacy by increasing CD8 T cell effector function.	Knudson et al. (73) National Cancer Institute, NIH

**Table 3:** Clinical trials of IL-15 in combination immunotherapy of cancer.

**IL-15 and combination agent**	**MTD or expansion dose/dosing schedule**	**Study population**	**Serious and notable adverse event**	**Maximum fold increase of NK cells**	**Best clinical response**	**References**
ALT-803 + nivolumab	20 μg/kg ALT-803 sc combination with IV nivolumab every 2 weeks	21 patients with metastatic non-small cell lung cancer	Grade 3 myocardial infarction. Injection site reaction.	3	6 PR, 10 SD	Wrangle et al. (74) Medical University of South Carolina, Health Hollings Cancer Center
*E. coli* rhIL-15 with haploidentical NK cell infusion	IL-15, 1.0 mcg/kg for 12 consecutive days IV with haploidentical NK cell infusion 2.0 mcg sc for 10 doses	42 patients: 26 IV and 16 sc with refractory acute myeloid leukemia	One patient died with cerebral infarct intracranial aspergilloma. 9 of 16 sc patients had CRS including fever, hypotension and in 5 of 9 concurrent neurotoxicity including one Grade 5.	NA	Of 15 IV patients: 6 CR and 2 Cri.	Cooley et al. (75) Masonic Cancer Center, University of Minnesota
*E. coli* rhIL-15 alemtuzumab	IL-15 sc Mon-Fri 0.5, 1.0, 2.0 mcg/kg/day for 2 weeks, followed by alemtuzumab 3, 10, 30 mcg/kg/day	8 patients with mature T cell malignancy	None	15	PR, CR elimination of leukemic T cells in each of 7 patients studied with leukemia	Miljkovic et al. (76)National Cancer Institute, NIH

*CIV, continuous intravenous infusion; d, day; IL-15, interleukin 15; IV, intravenous; Kg, kilogram; NA, not available; SC, subcutaneous; CR, complete response; PR, partial response; SD, stable disease; CRS, cytokine release syndrome*.

### Combination of IL-15 Plus Agonistic Anti-CD40

In rhesus macaques IL-15 by CIV at 20 mcg/kg/day for 10 days led to 80 to 100-fold increases in circulating effector memory CD8 T cells (41). Furthermore, rhIL-15 by CIV to patients with metastatic malignancy led to a mean 5.8-fold increase in the number of circulating CD8+, MHC class II^+^ cells (2, 45). However, this effect was not associated with evidence that the CD8 T cells manifested antitumor activity nor did it provide anticancer efficacy. In terms of CD8 T-cell function, γc cytokines such as IL-15 induced immunoregulatory SOCS checkpoint agents. IL-15 increased the expression of CIS, a checkpoint of NK cell mediated tumor immunity as well as SOCS1 that attenuates IL-15 receptor signaling by CD8+ CD44^hi^ memory T lymphocytes (77, 78). Furthermore, Sckisel et al. demonstrated that administration of gamma cytokines such as IL-2 and IL-15 led to paralytic depression of CD4 T cell that was mediated through transient expression of SOCS3 that inhibited STAT5B signaling (2, 79). This paralysis of CD4 helper T-cell activity inhibited the generation of tumor-specific CD8 T cells. It was demonstrated that CD4 helper cells' role could be provided by CD40 agonists (67, 68, 80–82). We showed in the TRAMP-C2 murine syngeneic tumor model that treatment with either an agonistic anti-CD40 antibody alone or IL-15 prolonged animal survival, however the combination of agonistic anti-CD40 with IL-15 produced markedly additive effects when compared with either agent alone (68). Neither agonistic anti-CD40 nor IL-15 augmented the number of tumor-specific CD8 T cells, whereas administration of the combination of IL-15 with an agonistic anti-CD40 antibody was associated with a 10-fold increase in the number of SPAS/SCNC 9H tetramer positive anti-TRAMP-C2 tumor specific CD8 T cells (68). Examination of this tumor system was extended by evaluating TRAMP-C2 administered on each flank with anti-CD40 administered intratumorally in one flank tumor and IL-15 administered systemically. In this model there was an abscopal effect obtained with reduction in the size of the tumor not injected with anti-CD40 beyond that mediated by IL-15 alone. A clinical trial is being initiated that utilizes an optimized intralesional anti-CD40 antibody in combination with CIV rhIL-15 (83, 84).

### Agents to Relieve IL-15 Induced Checkpoints on the Immune System to Augment IL-15 Action

IL-15 augments the expression of immune checkpoints TIGIT, TIM3, IL-10, as well as the expression of PD-1 on CD8 T cells (85, 86). Furthermore, IL-15 is required for the expression of a negative regulatory lymphocyte population that expresses CD122+ CD8+ (87). The combination of anti-PD-L1 with ALT-803 yielded additivity in murine tumor models (71–73, 88). Furthermore, trans-signaling with the RLI human IL-15 linked to the human IL-15Rα sushi domain augmented effector memory CD8 T-cell responses and enhanced antitumor activity of the PD-1 agonist (72). ALT-803 in combination with nivolumab in individuals with non-small cell lung cancer was associated with an objective response in 6 of 21 patients (74). To address checkpoints, we administered IL-15 in combination with antibodies to PD-L1 and cytotoxic lymphocyte antigen-4 (CTLA-4) in the CT26 and MC38 colon carcinoma and TRAMP-C2 prostatic cancer syngeneic tumor models (2, 69, 70). In these models IL-15 alone provided modest antitumor activity. The addition of either PD-L1 or CTLA-4 in association with IL-15 did not augment its efficacy. However, tumor-bearing mice receiving the combination of both anti-checkpoint antibodies with IL-15 manifested a significant prolongation of survival. In translation of this observation, a phase I trial in patients with refractory cancers has been initiated that involves rhIL-15 in combination with nivolumab and ipilimumab (NCT03388632).

### IL-15 in Combination Therapy With Anticancer Monoclonal Antibodies

As noted above, rhIL-15 administration led to dramatic increases in the number of activated NK cells, however such increases alone were not sufficient to produce antitumor efficacy probably because most tumors express self MHC class I molecules that interact with KIRs or NKG2A/CD94 that inhibit NK-effector function (2). Furthermore, there is lack of tumor cell identification and specific targeting by NK cells. The combination of IL-15 with tumor specific monoclonal antibodies has shown efficacy with a number of anticancer antibodies (Tables 2, 3) (55, 56, 58, 62, 64, 72). In preclinical trials IL-15 preparations have been reported to be of value in combination with *in vivo* administered anticancer monoclonal antibodies. In particular, IL-15 increased ADCC and antitumor activity when administered with anti-gp75, with B16F10 tumors (56) and with anti-CD20 with B-cell lymphomas (65). The combination of IL-15 with anti-PD1 or anti-PD-L-1 was more effective than with the individual agents alone (62, 69, 72, 75). In addition, an engineered fusion protein involving a soluble form of human IL-15Rα sushi with an antibody demonstrated antitumor responses (58). Furthermore, there was an enhanced ADCC and anti-breast cancer efficacy of cetuximab with a chimeric protein encompassing human IL-15 (62). We also investigated a combination therapy that involves IL-15 with rituximab in a syngeneic mouse model of EL4 transfected with human CD20 and with alemtuzumab (CAMPATH-1H) in a xenograft model of human adult T-cell leukemia (ATL) (69). IL-15 enhanced the therapeutic efficacy of both antibodies. This efficacy was dramatically reduced in FcRγ^−/−^ mice suggesting that IL-15 increased the ADCC of the anticancer monoclonal antibodies. Both NK cells and macrophages were critical elements of interacting effectors involved in the augmented ADCC and augmented therapeutic responses (71). Following interaction with macrophages there was induction of expression of FcRγIV critical for ADCC by the NK cells (2, 74). These results provided the scientific basis for a phase I trial of IL-15 combined with alemtuzumab (anti-CD52) for patients with ATL (NCT02689453) (2). Trials have also been initiated in patients with chronic lymphocytic leukemia with obinutuzumab in combination with rhL-15 (NCT03759184) and IL-15 with avelumab (anti-PD-L1) in patients with mature T-cell lymphoma (NCT03905135) and renal cell cancer (NCT04150562), and IL-15 with mogamulizumab (anti-CCR4, NCT04185220) in patients with ATL and cutaneous T-cell lymphoma.

## Conclusions

Despite their dramatic augmentation of NK cells and CD8 T cells, all IL-15 preparations administered as monotherapy in solid tumor patients with cancer have been ineffective probably due to counter-regulatory immunologic processes. In particular, there was inhibition of NK action by interaction of KIRs and NKG2A with self-class I MHC. There was parallel inhibition of CD8 T cells stimulated by IL-15 due to the induction of SOCS3 in CD4 helper T cells, thereby yielding “helpless” CD8 T cells (79). Furthermore, IL-15 induced checkpoints TIGIT, TIM3, IL-10, and PD-1 on CD8 T cells (85, 86). To circumvent these checkpoints combination trials that involve IL-15 with multiple anticancer agents are being performed. Our combination therapeutic trials include IL-15 with intralesional agonistic anti-CD40 to yield tumor specific CD8 T cells, IL-15 with the checkpoint inhibitors, anti-CTLA-4 and anti-PD-L1, and especially IL-15 with cancer directed monoclonal antibodies to increase their ADCC and anticancer efficacy. It is hoped with the use of these combination therapies that IL-15 will take a prominent role in the treatment of patients with metastatic malignancy.

## Author Contributions

TW designed, wrote, and edited the manuscript. SD, MM, and KC provided critical comments, concepts, and insights. All authors read and approved the manuscript prior to submission.

## Conflict of Interest

The authors declare that the research was conducted in the absence of any commercial or financial relationships that could be construed as a potential conflict of interest.
